# Volapük Not a Correct or Scientific World Language

**Published:** 1888-07

**Authors:** E. D. Babbitt

**Affiliations:** N. Y. College of Magnetics, 30 E. 14th st., N. Y.


### VOLAPÜK NOT A CORRECT OR SCIENTIFIC WORLD LANGUAGE.

All my life I have desired to see an ideal language developed on a scientific basis, a language built, as it were, on the foundations of the world, whose very structure would reveal the principles of things, beautiful, harmonious, sweet toned and founded on phonetic science. All things are fundamentally like all things and the sounds used in language should represent, as far as possible, the thing signified. Is this the case with Volapük, the much talked of world language, which, is being studied by several huundred thousand people ? I was greatly hoping it was, and when one of the New York papers called it "a jargon," I was grieved at the expression. When I came to study the language, however, my disappoint was great. Many of the words are harsh, preserving too much of the Teutonic style in which the author was reared, the language is but little briefer in its style of expression than the English, whereas an ideal language should be several times as brief, and all phonetic relations are entirely ignored. The beautiful masculine and feminine harmony of consonant sounds, and the graceful progression in pairs of the vowel sounds, seem to be entirely unknown to the author, Mr. Johann

Martin Schleyer, a simple hearted German priest who has succeeded in learning many languages but who has evidently no power to crystalise the principles of things into system. He has adopted the emperic method of using the vowels in alphabetical order instead of arranging them according to the law of sound ; constantly uses the German ü which hundreds of millions of the human race are unable to pronounce, makes no distinction between the sound of oo in pool, or oo in foot, omits the beautiful sound of u in tune, the dignified sound of a in awe, all, and several other vowels. As to the consonants, he makes j, contrary to all established use of language, sound like *sh.* and *c* like *j*, so that jip would be pronounced sheep and *cöc* judge. The pleasing sound of z as in zion, piazza, which is the masculine counterpart of *s*, he wholly omits, and calls it the two letters *ts* on the German plan. Some Cockneys find a difficulty in pronouncing the letter *r* after t, d, etc., and so he omits that and uses the incomparably worse combination *dl*, *tl*, as in dled, dlin, tlep, etc. This ignorance of phonetic harmonies and proprieties shows everywhere. It would seem evident enough that things which are sweet and lovely should have sweet sounds to represent them and that which is unlovely or rude should have harsher sounds, but Volapük is not philosophical enough for anything of that kind. The syllable *lu*, one of the sweetest of syllables, is used to signify " small insignificant, bad or contemptible," and is used with *fat* to form the word *lufat* or step-father. It would seem that stepfathers and stepmothers had a hard enough position without being dubbed with a *contemptible* name. Then such words as beautiful, musical have the rather ineuphoneous names jönik, musigik.

A true principle of language would require, that words most used should be brief and pleasant to utter. Here again, in various cases Mr. Schleyer exhibits his lack of thought. To say *is*, you must have it binom ; *are* is binoms ; *has* is labour ; gudikumo is better, *watch* is pokaglock, etc.

Euphony in language requires an equal balance of consonants and vowels like the Italian for instance Volapük has from 50 to 60 per cent more consonants than vowels, which accounts for its lack of euphony.

Volapük approaches scientific arrangement when it presents only one style of conjugation for verbs and one declension for nouns, but even here it cannot rise fully above the absurdities connected with all languages. What is the use of a series of cases to twist every noun out of its ordinary form ? What is the use of saying fat, fata, fate, fati, fatas, fatis, etc. for the different cases of the one word ? A noun can have an *s*, to pluralize it and a letter to denote possession but what more is needed ? If we wish to express *of* or *to* or any other preposition, put some letter or letters for it, and not pervert the word itself. What is the sense of having one form for the nominative (kimfal), and another for the objective (kimifal) The position in a sentence should always signify which is which. The subject is that which does something and should come first, the verb, or that which expresses what is done should come next, and the object, or that which is acted upon should come last. Then what earthly use is there in pluralizing a verb? The subject always tells the number and person and gender, and what more is wanted? The verb should be used either with or without a pronoun, such as he, she, it, they, a single letter signifying just which is meant. In Volapük, binom means *is* or *it is* or *he is*, three quite different things.

But the grandest of all features in a true world language will be the power of expression. It must be able to portray nice shades of meaning and be a true vehicle not only for the language of daily life, but for all exalted conceptions and for high spiritual, mental, scientific and philosophical ideas. Volapük seems to be especially deficient in power of expression, scarcely being up to ordinary English. Adjectives and adverbs in a true language must not include simply a comparative and a superlative degree but other increasing degrees and diminishing degrees, and progressive degrees, and moderate degrees and time degrees, etc.

It is thought by some that it will be used by business men who assemble from different nationalities to trade and do business together. So far as I have seen, however, it seems to be especially poor in commercial terms. It should have words to express whole phrases and thus shorten the drudgery of business intercourse, keeping of accounts etc.

I have said this much, desiring to check the thoughtless rush into a new language which is so lacking in conciseness, in euphony, in scientific character and in power of expression. Better tolerate the absurdities of the living languages of the day than to rush into new absurdities of a language which is not a living one.

*N. Y. College of Magnetics,*

30 *E.* 14*th st.*, *N. Y.* E. D. Babbitt, M. D.

Growth of the Brain.—The human brain reaches its greatest weight between the ages of fourteen and twenty in both sexes; after that it grows continually smaller through life. "While intelligence is rapidly increasing from twenty to sixty years of age, the brain is diminishing. The time that a man knows most is from seventy to eighty; but then his brain is smaller than when he was a boy between seven and fourteen, the age when he thought he knew the most.

